# Upregulation of *RHOXF2* and *ODF4* Expression in Breast Cancer Tissues

**DOI:** 10.22074/cellj.2015.8

**Published:** 2015-10-07

**Authors:** Golnesa Kazemi-Oula, Soudeh Ghafouri-Fard, Maryam Beigom Mobasheri, Lobat Geranpayeh, Mohammad Hossein Modarressi

**Affiliations:** 1 Department of Medical Genetics, Tehran University of Medical Sciences, Tehran, Iran; 2 Genetics Research Center, University of Social Welfare and Rehabilitation Sciences, Tehran, Iran; 3 Department of Medical Genetics, Shahid Beheshti University of Medical Sciences, Tehran, Iran; 4 Department of Surgery, Sina Hospital, Tehran University of Medical Sciences, Tehran, Iran

**Keywords:** Breast Cancer, Cancer-Testis Antigen, *ODF4*, *RHOXF2*

## Abstract

**Objective:**

During the past decade, the importance of biomarker discovery has been highlighted in many aspects of cancer research. Biomarkers may have a role in early detection of cancer, prognosis and survival evaluation as well as drug response. Cancer-testis antigens (CTAs) have gained attention as cancer biomarkers because of their expression in a wide variety of tumors and restricted expression in testis. The aim of this study was to find putative biomarkers for breast cancer.

**Materials and Methods:**

In this applied-descriptive study, the expression of 4 CTAs, namely acrosin binding protein (*ACRBP*), outer dense fiber 4 (*ODF4*), Rhox homeobox family member 2 (*RHOXF2*) and spermatogenesis associated 19 (*SPATA19*) were ana- lyzed at the transcript level in two breast cancer lines (MCF-7 and MDA-MB-231), 40 invasive ductal carcinoma samples and their adjacent normal tissues as well as 10 fibroadenoma samples by means of quantitative real-time reverse transcription polymerase chain reaction (RT-PCR).

**Results:**

All four genes were expressed in both cell lines. Expression of *ODF4* and RH- OXF2 was detected in 62.5% and 60% of breast cancer tissues but in 22.5 and 17.5% of normal tissues examined respectively. The expression of both *RHOXF2* and *ODF4* was upregulated in cancerous tissues compared with their normal adjacent tissues by 3.31 and 2.96-fold respectively. The expression of both genes was correlated with HER2/neu overexpression. *RHOXF2* expression but not *ODF4* was correlated with higher stages of tumors. However, no significant association was seen between expression patterns and estrogen and progesterone receptors status.

**Conclusion:**

*ODF4* and *RHOXF2* are proposed as putative breast cancer biomarkers at the transcript level. However, their expression at protein level should be evaluated in future studies.

## Introduction

Currently, there is emerging data on tumor associated antigens which are differentially expressed in cancer tissues and can be used as cancer biomarkers. The tremendous achievements in the field of cancer biomarker discovery have enhanced the efficiency of early cancer detection and treatment (1). Cancer-testis antigens (CTAs) are a group of tumor-associated antigens with more than 150 members which are preferentially expressed in gametogenic tissues and aberrantly expressed in tumors (1). The expression of several members of this family has been assessed in various cancers including breast cancer. National Cancer Institute of the United States has placed two CTAs, namely MAGE-A3 and NY-ESO-1, into the top 10 category of the Project for the Prioritization of Cancer Antigens (2). Since the testis is assumed as an immune privileged site, if testis-specific genes are expressed in other tissues, they can elicit an immune response.

We have previously analyzed expression of some members of this family in breast cancer and reported TSGA10 and FBXO39 as cancer biomarkers and candidates for immunotherapy of breast cancer (3, 4). In this study we aimed to assess expression of four CTAs, namely [acrosin binding protein (*ACRBP*), outer dense fiber 4 (*ODF4*), Rhox homeobox family member 2 (*RHOXF2*) and spermatogenesis associated 19 (*SPATA19*)] in two breast cancer cell lines, 40 invasive ductal carcinoma samples and their adjacent normal tissues as well as fibroadenoma samples. The selection of these CTAs was based on previous work demonstrating their expression in a wide variety of tumors except for breast cancer tissues which were not previously studied. *ACRBP* is a testisselective gene which has been shown to be expressed in a variety of cancers at the transcript level and in ovarian cancer at the protein level. In addition, it has elicited spontaneous humoral responses in some cancer patients (5). These responses make *ACRBP* a putative candidate for active immunotherapy. *SPATA19* is proposed as a possible target for cancer immunotherapy and a novel marker for early detection of basal cell carcinoma of skin and prostate cancer. In addition, it has a mitochondria-targeting signal which can be recruited in mitochondrial targeting strategies for treatment of cancer (6, 7). *ODF4* is another testis-specific gene whose overexpression has been detected in chronic myeloid leukemia patients (8). Furthermore, its alternative splice variants have been seen in testis of a prostate cancer patient (9). *RHOXF2* is a CTA expressed in a wide variety of cancer cell lines and tumor samples. Knockdown of *RHOXF2* has decreased the growth of a gastric cancer cell line HGC27 and its overexpression in HF6 cells has rapidly induced leukemia in transplanted mice. So it has been concluded that *RHOXF2* has role in cell transformation (10). In addition, it is a stem cell marker (11) which has a role in cell to cell contact (12).

## Materials and Methods

### Tissue samples

This applied-descriptive study was approved by the Ethics Committee of Tehran University of Medical Sciences (21/5604). Forty invasive ductal carcinoma of breast and their adjacent normal tissues along with 10 fibroadenoma samples were taken from patients in Sina hospital under the protocols of the Ethics Committee. Normal testis tissue was taken from a prostate cancer patient following orchiectomy. Tissues to be subjected for RNA extraction were frozen in liquid nitrogen. Informed consent was obtained from all adult human participants.

### Immunohistochemical analysis

Immunohistochemical (IHC) analysis was performed on 4 μm thick paraffin-embedded formalin- fixed tissue sections. IHC for HER2 was performed using the HercepTest kit according to the manufacturer’s protocol (Dako, Denmark). In brief, sections were deparaffinized and rehydrated in graded alcohols. The slides were then incubated with pre-diluted anti-HER2 antibody, washed in phosphate buffered saline (PBS) and incubated with horseradish peroxidase-conjugated secondary antibody. Estrogen receptor (ER) and progesterone receptor (PR) status was checked with 1D5 and PGR-1A6 antibodies respectively (Dako, Glostrup, Denmark). The HER2/neu expression was scored based on the degree of membrane staining according to previous guidelines (13). Samples with HER2/neu scores of 2 or more were regarded as positive. Samples with nuclear staining for ER and/or PR in more than 10% of the tumor cells were considered as ER and/or PR positive. P53 status was evaluated with a commercial antibody designed to detect the N-terminal of P53 (Dako, Denmark).

### Cell culture

The human breast cancer cell lines MDAMB- 231 and MCF-7 were purchased from Pasteur Institute of Iran and cultured according to the manufacturer’s instruction. Cells were cultured in RPMI-1640 medium (Sigma Aldrich, USA) supplemented with 10% fetal bovine serum, 100 U/ml penicillin, and 100 μg/ml streptomycin. The cells were plated and incubated in 5% CO_2_/95% humidity at 37˚C.

### RNA extraction and quantitative real-time reverse transcription polymerase chain reaction (RT-PCR)

Total RNA was extracted from tissue samples and cells using TriPure Isolation Reagent (Roche Applied Science, Germany) as instructed by the manufacturer. RNA was analyzed by Thermo Scientific NanoDrop™ 1000 Spectrophotometer to check its purity and concentration, and electrophoresd to confirm its integrity. One μg of RNA was used for cDNA synthesis by using Fermentas RevertAidTM H Minus First Strand cDNA Synthesis Kit (Fermentas, Canada). Synthesized cDNA was then checked spectrophotometrically to estimate its concentration. Quantitative realtime reverse transcription polymerase chain reaction (RT-PCR) reaction was carried out on a rotor gene 6000 corbette detection system using AccuPower ® 2X Greenstar qPCR Master Mix (BIONEER, USA). Normal testis cDNA was used as a positive control for gene expression. Thermal cycling conditions were an initial activation step for 5 minutes at 95˚C followed by 40 cycles of denaturation step for 10 seconds at 95˚C, annealing step for 10 seconds at 60˚C and extension step for 15 seconds at 72˚C. No template control (NTC) consisting of H_2_O was included in each run. HPRT gene was used as normalizer (6). Primer sequences are listed in table 1. Melting curve analysis was performed to verify specificity of PCR products. In addition, PCR products were electrophoresed on 2% agarose gel to confirm product sizes and specificity.

### Statistical analysis

Fold changes in gene expression were calculated by LinRegPCR (2) and Relative Expression Software Tool-RG©-version 3 (QIAGEN, Korea). The amounts of mRNAs in the tissues, standardized to the HPRT mRNA, were calculated as follows: -ΔCT=-[CT Gene of interest-CT HPRT]. The level of statistical significance was set at P<0.05. Statistical analyses were performed using SPSSv.15.0.1 (SPSS Inc., Chicago, IL). To compare clinical and demographic characteristics between patients expressing the mentioned genes with those not, Mann-Whitney or t test (considering the presence of normal distribution) was used. Normality of quantitative data was tested using Kolmogorov- Smirnov test.

**Table 1:** Sequence of primers used in this study


Primer	Sequence

*HPRT*	F: 5΄-CCTGGCGTCGTGATTAGTGAT-3΄
	R: 5΄-AGACGTTCAGTCCTGTCCATAA-3΄
*RHOXF2*	F: 5΄-GCTACTGCCCCACCATGACC-3΄
	R: 5΄-ATGGACTCGAAGCGCACATC-3΄
*ODF4*	F: 5΄-GCTTATCCTATACTTCAAATGCG-3΄
	R: 5΄-GCCAGGAGTTCAGAAAAGATTACAC-3΄
*SPATA19*	F: 5΄-CAAACCAGAGCCAAGAGGTCC-3΄
	R: 5΄-GGATATGCTTCGTCTCACCTGC-3΄
*ACRBP*	F: 5΄-CTTCCTTCCCTCACTCCTGAAGG-3΄
	R: 5΄-GCCGTGGGTTGCACGGAGAC-3΄


## Results

### Demographic and clinical data of patients

Demographic data of patients are summarized in table 2. IHC analyses showed that 62.5, 60 and 42.5% of samples were ER, PR and P53 positive respectively. The intensity of staining for HER2/ neu was 1+, 2+, 3+ and 4+ in 45, 12.5, 17.5 and 25% of samples respectively.

**Table 2:** Demographic and clinical data of patients


Age (mean ± SD)	51.28 ± 10.56 (25-68)
Menarche age (Y)	12.32 ± 1.63
Menopause age (Y)	52.55 ± 1.38
Positive family history for cancer (%)	54
Cancer stage (%)	
0	2.5
I	10
II	27.5
III	10
IV	50


### Expression of ACRBP, ODF4, RHOXF2 and SPATA19 in MCF-7 and MDA-MB-231 cell lines

**Fig.1:**
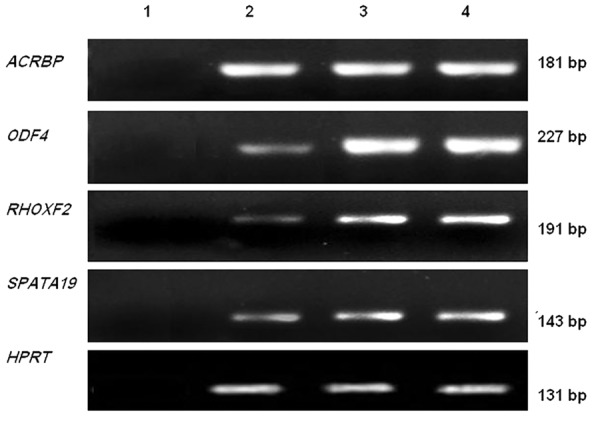
Expression of *ACRBP*, *ODF4*, *RHOXF2* and *SPATA19* in MCF-7 and MDA-MB-231. Lane 1; Negative control, lane 2; MCF-7, lane 3; MDA-MB-231 and lane 4; Testis sample.

Real-time PCR showed that both cell lines expressed the 4 mentioned genes (Fig .1).

### The relative expression ratios of *ODF4* and *RHOXF2* in cell lines and testis tissue

**Fig.2:**
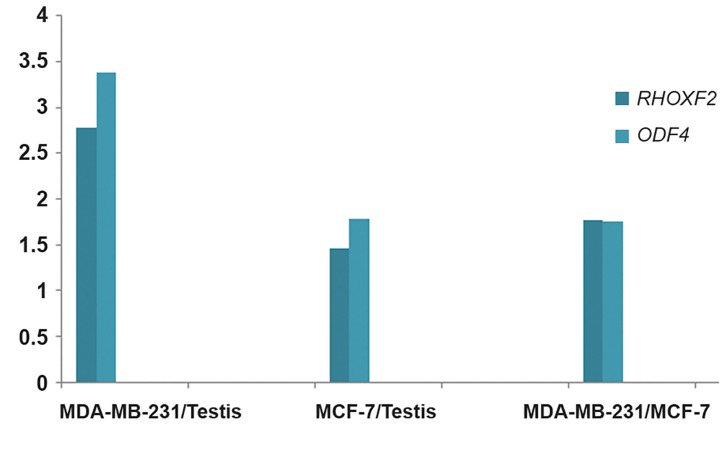
Relative expression ratios for *RHOXF2* and *ODF4* in MDAMB- 231 and MCF-7 cell lines compared with each other and with testis.

The expression of *ODF4* was up-regulated in MCF-7 and MDA-MB-231 compared with normal testis sample by 1.77and 3.38-fold respectively. The expression of *RHOXF2* was also upregulated in MCF-7 and MDA-MB-231 compared with normal testis sample by 1.46and 2.78-fold respectively (Fig .2).

### The relative expression ratios of *ODF4* and *RHOXF2* in MCF-7 and MDA-MB-231 cell lines

Real time RT-PCR results showed that *ODF4* and *RHOXF2* expression were significantly higher in MDA-MB-231 than MCF-7 (P value< 0.001, Fig .2).

### Expression of ACRBP, ODF4, RHOXF2 and SPATA19 in fibroadenoma samples

**Fig.3:**
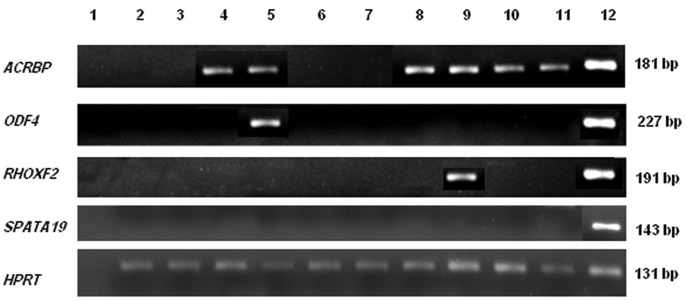
Expression of *ACRBP*, *ODF4*, *RHOXF2* and *SPATA19* in fibroadenoma samples. Lane 1; Negative control, lanes 2-11; Fibroadenoma samples and lane 12: Testis sample.

*ACRBP*, *ODF4* and *RHOXF2* were expressed in 60, 10 and 10% of fibroadenomas respectively. None of the fibroadenoma samples showed SPATA19 expression (Fig .3).

### Expression of ACRBP, ODF4, RHOXF2 and SPATA19 in breast tissue samples

**Fig.4:**
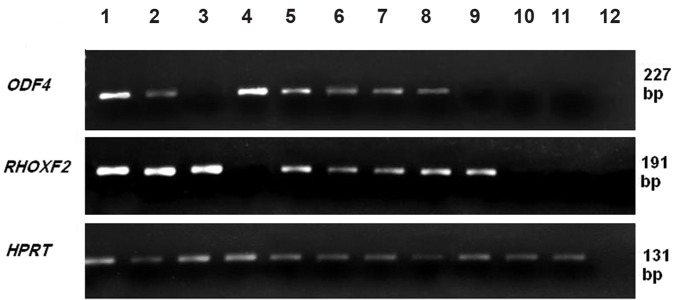
Expression of *ACRBP*, *ODF4*, *RHOXF2* and *SPATA19* in breast cancer and their adjacent normal tissues. Lane 1; Testis sample, lanes 2-9; Cancer samples, lanes 10, 11; Adjacent normal tissues and lane 12; Negative control.

**Fig.5:**
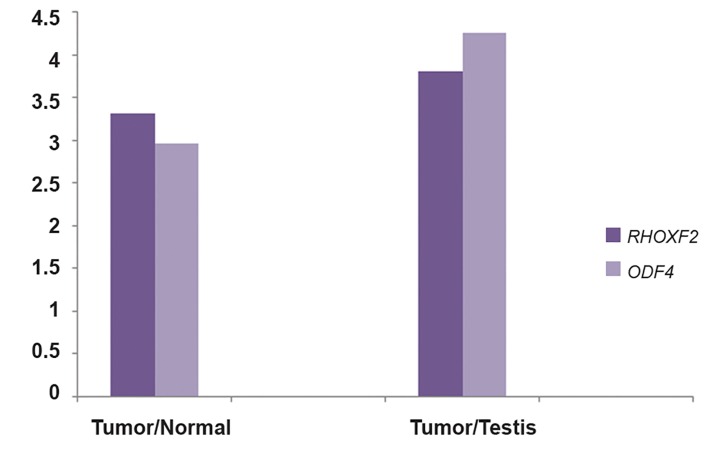
Relative expression ratios for *RHOXF2* and *ODF4* in tumor tissues and adjacent normal tissues.

*ACRBP* was expressed in normal breast and fibroadenoma samples and was therefore excluded from further analysis. *SPATA19* showed no significant difference in cancerous versus normal tissues. *ODF4* and *RHOXF2* expressions were detected in 62.5 and 60% of breast cancer tissues but also in 22.5 and 17.5% of normal tissues examined respectively (Fig .4). A significant up-regulation of *RHOXF2* and *ODF4* genes was observed in cancer tissues compared with normal adjacent tissues by 3.31and 2.96-fold respectively (P value<0.001, Fig .5). The expression of both genes was correlated with HER2/neu overexpression. *RHOXF2* expression but not *ODF4* was correlated with higher stages of tumors. There was no significant relationship between expression of these genes and ER, PR and P53 status.

## Discussion

CTAs are considered as cancer biomarkers and putative candidates for cancer immunotherapy as well as immunoprevention in high risk individuals (14). Expression analysis of CTAs in breast cancer patients may pave the way to find targets for polyvalent vaccines (14). CTAs are expressed in breast cancer tumors especially in the triple negative subtype (15-17). As therapeutic options are limited for this cancer subtype, CTAs can provide novel therapeutic approaches for these patients. Some CTAs such as *ODF4* have been shown to be expressed only in testis among normal tissues. For some others such as *ACRBP*, the expression has been detected in other normal tissues albeit at a lower level than testis. CTAs with a more restricted expression pattern are more suitable targets for cancer immunotherapy. Among the four genes analyzed in this study, *ODF4* and *RHOXF2* showed overexpression in cancerous tissues compared with normal adjacent tissues. These two genes are proposed as putative cancer biomarkers which can be used in combination with other biomarkers to differentiate cancerous versus normal tissues. ODF proteins are responsible for maintaining the sperm tail (18). Some of them have been shown to be elements of the centrosome matrix (18). As the centrosome has an essential role in efficient mitosis, elevated expression of ODF genes in cancer cells may facilitate their rapid proliferation. Considering the role of *RHOXF2* cell transformation (10) and cell to cell contacts (12), in addition to its up-regulation in breast cancer tissues (the present study), it may participate in the process of tumorigenesis.

In this study, we analyzed expression of 4 CTAs in an ER positive breast cancer cell line (MCF-7) and an ER negative one (MDA-MB-231). MCF-7 is considered to have a relatively benign phenotype compared with MDA-MB-231 which is a highly invasive metastatic cell line. The expression analysis of genes involved in cell migration, invasion and metastasis as well as anti apoptotic genes has indicated that MDA-MB-231 cells have a much more malignant molecular profile than MCF-7 cells (19). Previously we reported that RHOXF1 is overexpressed in MDA-MB-231 compared with MCF-7 (11). The considerably higher expression of *RHOXF2* and *ODF4* in MDA-MB-231 than in MCF-7 implies a role for these genes in malignant phenotype.

Cellular microenvironment has an essential role in breast tumorigenesis. In a previous study, whole genome expression pattern has been analyzed in histologically normal tissues adjacent to breast tumor to find whether it is altered in the adjacent normal tissues by the tumor and how much this is different from breast reduction tissue. It has been shown by whole genome microarray analysis that there is no significant alteration in gene expression of morphologically normal tissue adjacent to breast carcinomas and breast reduction tissue (20). However, a more recent study using RNA-Seq data from breast cancer and adjacent normal tissue has revealed complete differential gene expression between the two especially in Fos, Jun and TGF beta pathways which are active in the adjacent normal tissues. It has therefore been concluded that tissue adjacent to a primary breast cancer is not normal when compared to healthy breast tissue (20). Further research should compare expression of CTAs including *ODF4* and *RHOXF2* in breast reduction tissues and normal adjacent tissues to measure the alterations in gene expression from healthy normal to normal adjacent-to-tumor to tumor tissues. Another recent study has shown that cancer stem cell markers are augmented in normal tissue adjacent to triple negative breast cancer tissue (21). It has also been hypothesized that CTAs in addition to being a feature of gametogenesis, are stem cell markers (22). Future studies should therefore focus on evaluation of *ODF4* and *RHOXF2* immunogenicity to find out whether they can be used in active immunotherapy. Evaluation of their expression at the protein level is also suggested which was a limitation of our study.

## Conclusion

In this study we have analyzed the expression of four CTAs in breast cancer tissues and their histologically normal appearing adjacent tissues and found differential expression for two genes. Differential expression pattern of these genes in normal versus cancerous tissues implies their potential as cancer biomarkers. However, more experiments at the proteome level and with a larger cohort of patients are needed to evaluate this finding.
